# Management of chronic rheumatic diseases in women 18–45 years of age in Asia Pacific: insights from patient and clinician surveys

**DOI:** 10.1007/s00296-022-05206-0

**Published:** 2022-09-26

**Authors:** Yoshiya Tanaka, Claire Barrett, Yuji Hirano, Kei Ikeda, Kathy Paizis, Azusa Sameshima, Irina Mountian, Priscilla C. Wong

**Affiliations:** 1 University of Occupational and Environmental Health, Japan, Kitakyushu, Japan; 2 Redcliffe Hospital, Redcliffe and University of Queensland, St Lucia, Australia; 3 Toyohashi Municipal Hospital, Toyohashi, Japan; 4 Chiba University Hospital, Chiba, Japan; 5 Austin Health, and Mercy Hospital for Women, Heidelberg, and Western Health, St Albans, Australia; 6 University of Toyama, Toyama, Japan; 7 UCB Pharma, Brussels, Belgium; 8 The Chinese University of Hong Kong, Hong Kong, HK Hong Kong

**Keywords:** Chronic rheumatic diseases, Family planning, Patients, Pregnancy, Survey, Tumour necrosis factor inhibitors

## Abstract

**Objective:**

Perspectives of women aged 18–45 years with chronic rheumatic diseases (CRD), and clinicians, in the Asia-Pacific (APAC) region are reported.

**Methods:**

Online surveys were completed by women, pregnant in the past 2–5 years, with moderate to severe rheumatoid arthritis (RA), psoriatic arthritis (PsA), axial spondyloarthritis (axSpA), and rheumatologists, obstetricians, orthopaedic surgeons who medically manage CRDs.

**Results:**

Among 210 (RA 122, PsA 48, axSpA 40) patients, 52% (*n* = 109/210) delayed their decision to have children, most commonly due to concerns of passing on disease to offspring. 33% (*n* = 70/210) discussed family planning with a healthcare professional at diagnosis. Patients most often initiated discussions. 94% (*n* = 193/205) stopped treatment around pregnancy due to fear of fetal harm. 66% (*n* = 139/210) of patients felt they did not receive all relevant information on the impact of CRDs and treatment on pregnancy.

Among 335 clinicians who participated, 82% (*n* = 143/174) of rheumatologists, 86% (*n* = 72/84) of obstetricians and 43% (*n* = 33/77) of orthopaedic surgeons agreed good disease control during pregnancy was their primary goal. 69% (*n* = 120/174) of rheumatologists were ‘very comfortable’ with prescribing tumour necrosis factor inhibitors (TNFi) for women aged 18–45 years. Comfort levels generally decreased with the onset of family planning. More obstetricians and orthopaedic surgeons supported avoiding TNFi during pregnancy than rheumatologists (40% [*n* = 34/84]/38% [*n* = 29/77] versus 16% [*n* = 28/174]). Access to more TNFi safety data during pregnancy was considered paramount for increasing clinician comfort.

**Conclusions:**

Patients and physicians need current information and multidisciplinary discussions for improved management of CRD in women in APAC.

**Supplementary Information:**

The online version contains supplementary material available at 10.1007/s00296-022-05206-0.

## Introduction

In women with chronic rheumatic diseases (CRD) such as rheumatoid arthritis (RA), axial spondyloarthritis (axSpA) and psoriatic arthritis (PsA), disease onset, diagnosis and treatment initiation often overlap with their peak reproductive years (18–45 years) [1, 2]. The availability of successful treatment options means that many patients now have improved physical function and quality of life, and may like to consider starting a family [1].

Current EULAR recommendations suggest addressing family planning in all patients of reproductive age, and a multidisciplinary approach to managing these patients [3]. However, most physicians do not proactively discuss family planning with their female patients in this age group. A survey of physicians in the U.S. and in Europe found that only 32–‍56% of them initiated discussions on family planning with their patients [1].

In patients who might be planning to start a family, their ability to conceive may be affected by their disease [4], and they may have worries regarding the impact of disease and treatments on pregnancy. In a 2017 survey conducted in patients in the U.S. and Europe, 54% of women with CRD admitted that they had delayed their decision to start a family, and that their main concern was passing on health issues to the child [5].

During pregnancy, it is important to keep good disease control as active disease at this time can lead to complications and poor pregnancy outcomes, for example preterm birth and growth restriction [6]. Among the biologic disease-modifying antirheumatic drugs (bDMARDs), most tumour necrosis factor inhibitors (TNFi) such as adalimumab, etanercept, infliximab and golimumab may be considered for use in the first and second trimester of pregnancy while certolizumab pegol, an Fc-free TNFi which has little or no placental transfer, may be considered during all three trimesters of pregnancy if clinically needed [3, 7–9]. Despite the recommendations [3, 7–10], a previous survey of clinicians in the U.S. and Europe reported a decline in confidence in prescribing TNFi with the onset of family planning as they were concerned about the adverse events (AEs) of the treatments [11]. Inadequate knowledge and general fear towards the compatibility of medications with pregnancy and breastfeeding could result in clinicians unnecessarily discontinuing therapy, thus putting patients at risk of relapse and potential adverse pregnancy outcomes [12–14].

In the U.S. and Europe, women have reported several fears and misconceptions on these topics and their clinicians also have uncertainties and concerns about the risks and benefits of TNFi in women 18–45 years of age [5, 11]. An online survey was conducted in the APAC region to explore the perspectives of women aged 18–45 years with CRD around disease management, family planning and whether they received support from their clinicians. A separate survey explored clinicians’ attitudes towards treating these women with TNFi. Clinicians were asked about their comfort levels with prescribing TNFi treatment in women 18–45 years of age, decisions around the use of TNFi prior to, during and after pregnancy, and concerns around AEs associated with TNFi during pregnancy.

## Methods

### Participants

#### Patient survey

Participants included women diagnosed with CRD (either RA, PsA or axSpA) across Australia, Japan, Hong Kong and Taiwan. Eligible patients were women 18 − 45 years of age with self-reported moderate to severe RA, PsA or axSpA. Patients were not pregnant at the time of enrolment but had been pregnant in the past 2–5 years and had used medication at some point in their disease history.

#### Clinician survey

Participants included rheumatologists and obstetricians from Australia, Japan, Hong Kong and Taiwan, and orthopaedic surgeons from Japan only. Orthopaedic surgeons from Japan were included in this survey as they treat patients with CRD and prescribe both conventional synthetic DMARDs (csDMARDs) and bDMARDs. Eligible rheumatologists and orthopaedic surgeons were medically managing female patients aged 18–45 years who had a CRD, such as RA, PsA or axSpA. Eligible obstetricians were co-managing ≥ 1 female patient with CRD aged 18–45 years who had been prescribed TNFi in the past 3 years.

### Study design

#### Patient survey

Twenty-minute online surveys (37 questions in total, including screening questions) were conducted in patients from September to October 2018, by the market research company Hummingbird Insight (Sydney, Australia). Patients were recruited from consumer panels, by Hummingbird Insight (Sydney, Australia). Since this was a market research study, prior approval of the protocol by an ethics committee was not required. As the market research company involved is based in Australia, the study was conducted in accordance with Australian market research guidelines, including the obtaining of informed consent and adherence to ethical reporting standards. The market research company conducting the study also acts in accordance with the appropriate codes of conduct regarding anonymity and confidentiality and are also fully compliant with the Data Protection Act.

The patient questionnaire, developed by UCB Pharma, was adapted from a previous global survey developed to evaluate the perspectives of women in the U.S. and Europe. [5] It was designed to identify how they balanced family planning and the treatment needed to manage their condition. The patients were also asked about different topics such as information received from HCPs, pre-conception planning, treatment around pregnancy and breastfeeding. The survey was developed in English and was delivered in the local language. Translations of the questionnaire were conducted by Hummingbird Insight (Sydney, Australia). The full patient questionnaire can be found in Supplementary Table 1.

#### Clinician survey

Online surveys (11 questions in total, including screening questions) were conducted in clinicians from September to October 2018, by the contract research organisation IQVIA (Sydney, Australia). A panel of clinicians were contacted and invited to complete the online survey, by IQVIA (Sydney, Australia). Since this was a market research study, prior approval of the protocol by an ethics committee was not required. As the market research company involved is based in Australia, the study was conducted in accordance with Australian market research guidelines, including the obtaining of informed consent and adherence to ethical reporting standards. The market research company conducting the study also acts in accordance with the appropriate codes of conduct regarding anonymity and confidentiality and are also fully compliant with the Data Protection Act.

The clinician questionnaire, developed by UCB Pharma, was adapted from a global survey developed to evaluate the perspectives of clinicians in the U.S. and Europe [11]. It was designed to assess their comfort levels with prescribing TNFi treatment in women 18–‍45 years of age, decisions around the use of TNFi prior to, during and after pregnancy, and concerns around AEs associated with TNFi during pregnancy. The questionnaire completed by the clinicians (rheumatologists, obstetricians and orthopaedic surgeons) were the same, except for screening questions. The survey was developed in English and was delivered in the local language. Translations of the questionnaire were conducted by IQVIA (Sydney, Australia). The full clinician questionnaire can be found in Supplementary Table 2.

### Statistical analysis

Data were summarised descriptively for APAC overall (Australia, Japan and Hong Kong/Taiwan) and individually. Data were reported as proportions of patients or clinicians. No statistical analysis was conducted.

## Results

### Patient survey

#### Participant demographics and disease characteristics

A total of 210 women met the inclusion and exclusion criteria and completed the survey. Among them, 108 (51%) were from Australia, 68 (32%) from Japan and 34 (16%) from Hong Kong/Taiwan (Supplementary Table 3). In total, 58% (*n* = 122) had a diagnosis of RA, 23% (*n* = 48) had PsA and 19% (*n* = 40) had axSpA. Most patients (63%; *n* = 133) were 31–‍40 years old, and overall, 77% (*n* = 162) reported their CRD to be of moderate severity. More than half (54%; *n* = 113) of the patients had been pregnant in the last 2 years, and 72% (*n* = 151) of patients had been diagnosed before becoming pregnant. Around one-third (37%; *n* = 78) of the patients used TNFi peri-pregnancy.

#### Overall information gaps

In total, 66% (*n* = 139/210) of patients felt they did not receive all the relevant information they needed on the impact of rheumatic disease and treatment on pregnancy and health, from their HCPs. Patients reported having liked more information around the impact of the disease and treatment on themselves and their baby (Supplementary Fig. 1).

#### Family planning

**Fig. 1:**
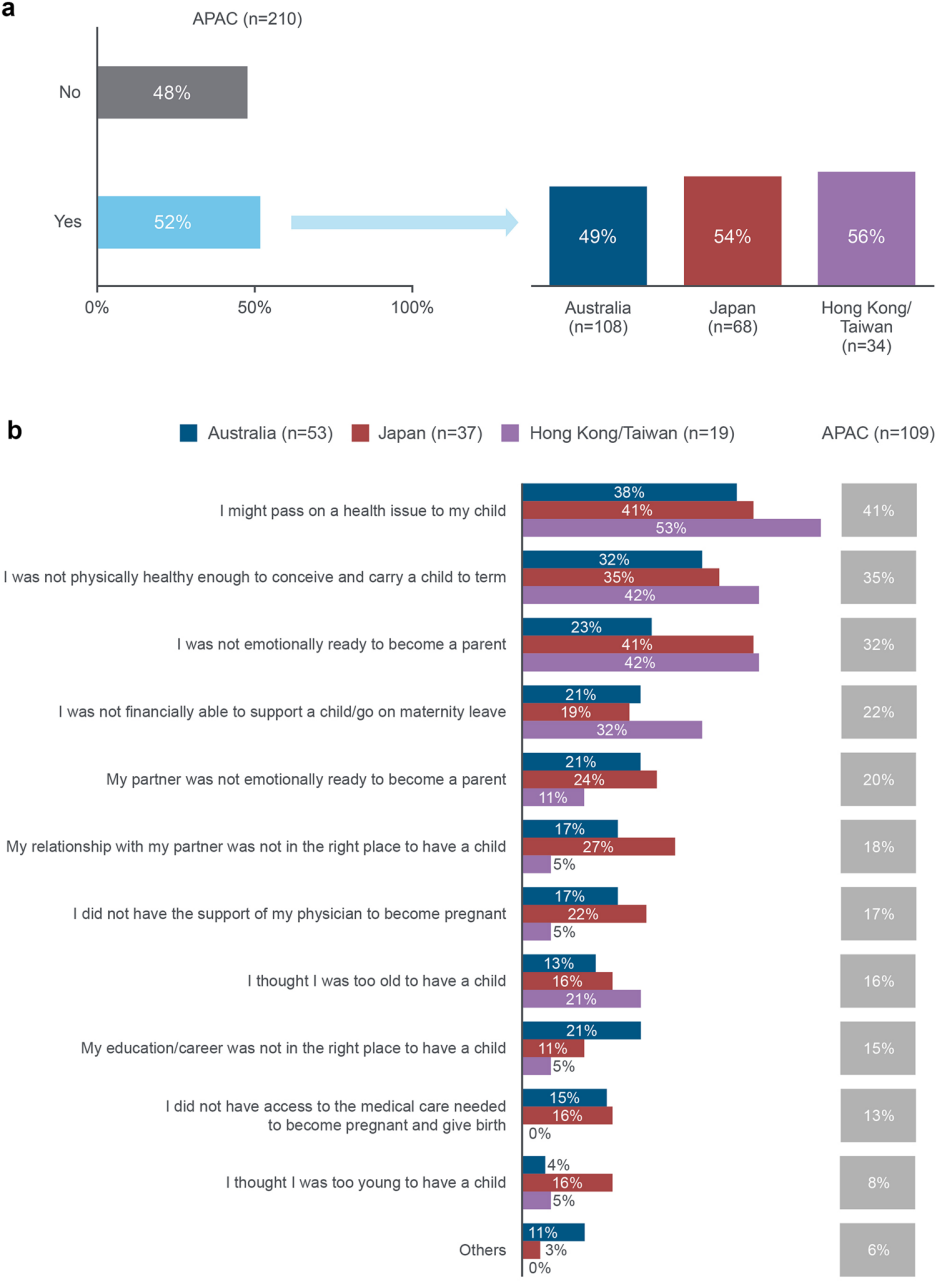
Proportion of women with CRD and their fears that that delayed their decision to become a mother. a) Proportion of women with CRD that delayed their decision to become a mother b) Fears of women with CRD that delayed their decision to become a mother. (a) ‘Did you have any concern that delayed your decision to become a mother for your most recent pregnancy?’ (b) ‘Which (if any) of the following concerns delayed your decision to become a mother for your most recent pregnancy?’ Results reported for those who indicated ‘yes’ in the previous question. Multiple answers were possible. APAC: Asia–Pacific (Australia, Japan and Hong Kong/Taiwan); CRD: chronic rheumatic disease

Fewer women in Australia (31%; *n* = 33/108) were actively trying to get pregnant prior to their most recent pregnancy, compared with Japan (53%; *n* = 36/68) and Hong Kong/Taiwan (44%; *n* = 15/34). Overall, 52% (*n* = 109/210) of patients reported delaying their decision to have children (Fig. 1a). Their most common concern was passing on a health issue to their child (Fig. 1b).

Pregnancy planning was first discussed at the time of diagnosis in 33% (*n* = 70/210) of patients (Australia: 27% [29/108]; Japan: 34% [*n* = 23/68]; Hong Kong/Taiwan: 53% [*n* = 18/34]); 32% (*n* = 68/210) at the time of treatment initiation (Australia: 39% [*n* = 42/108]; Japan: 28% [*n* = 19/68]; Hong Kong/Taiwan: 21% [*n* = 7/34]) and 30% (*n* = 64/210) during a regular visit (Australia: 31% [*n* = 34/108]; Japan: 32% [*n* = 22/68]; Hong Kong/Taiwan: 24% [*n* = 8/34]). Discussions on family planning with HCPs were most often initiated by patients (45% of the time; *n* = 95/210), followed by HCPs (35%; *n* = 73/210) and by their partner (17%; *n* = 35/210). Overall, 40% (*n* = 84/210) of patients had a treatment plan discussed and aligned between HCPs, 30% (*n* = 63/210) had a treatment plan but it was not aligned between HCPs, and 30% (*n* = 63/210) did not have a treatment plan.

#### Pregnancy

**Fig. 2:**
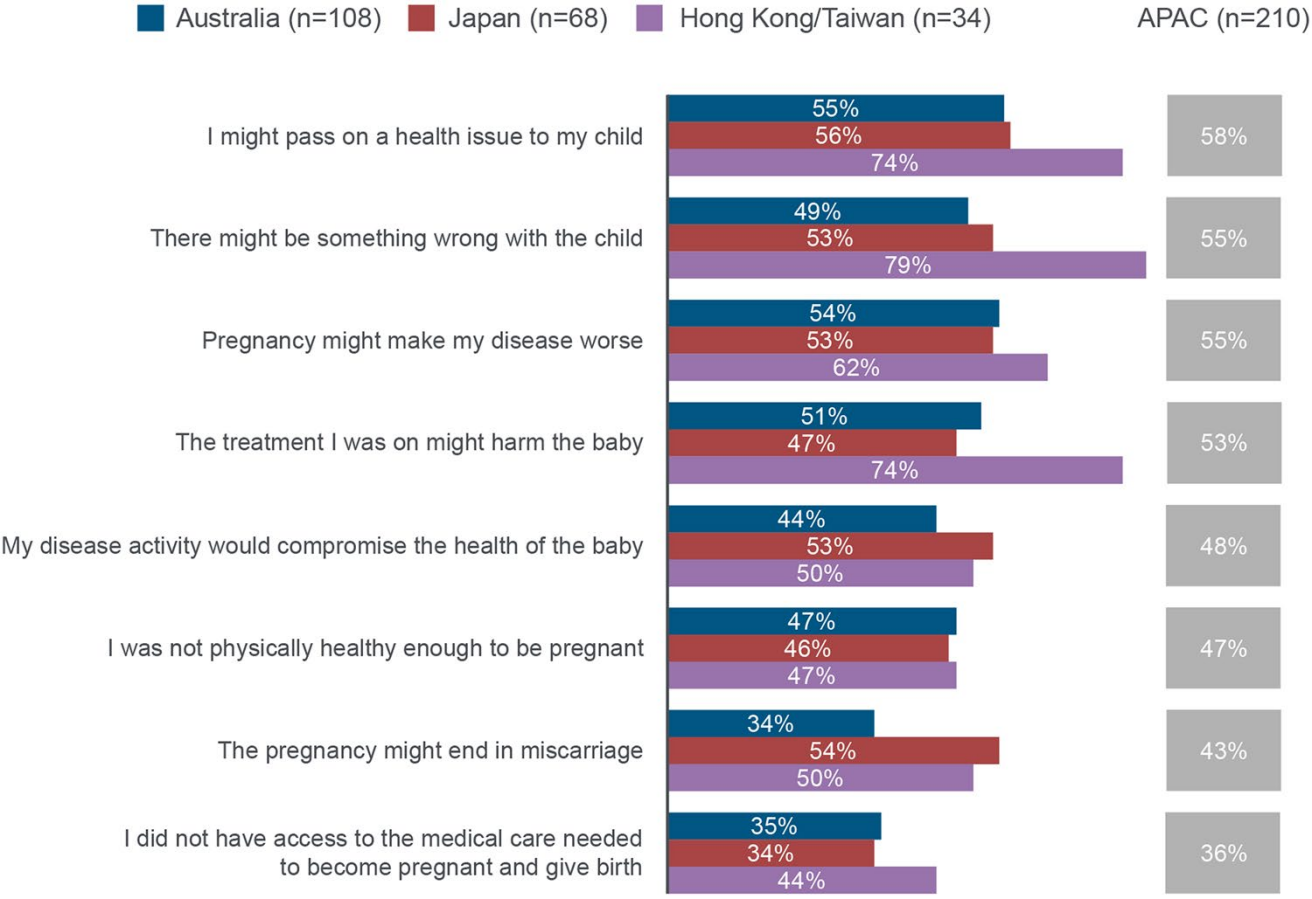
Concerns of women upon discovering they were pregnant. ‘At the time you discovered you were pregnant, to what extent did you experience any of the following concerns?’ Results reported for those who indicated a score of 4/5 for concern in the questions (1 representing ‘not at all concerned’ and 5 representing ‘very concerned’). APAC: Asia–Pacific (Australia, Japan and Hong Kong/Taiwan)

Upon discovering they were pregnant, patients’ main concern was passing on a health issue to the child (Fig. 2). During pregnancy, 52% (*n* = 109/210) of patients discussed their CRD condition with a rheumatologist versus obstetrician/gynaecologist (47%; *n* = 98/210), general practitioner (GP; 33%; *n* = 69/210) and midwife (26%; *n* = 54/210). Disease improvement during pregnancy was reported in 55% (*n* = 115/210) of patients, 27% (*n* = 57/210) reported stable disease and 18% (*n* = 38/210) experienced worsening of disease activity.

**Fig. 3:**
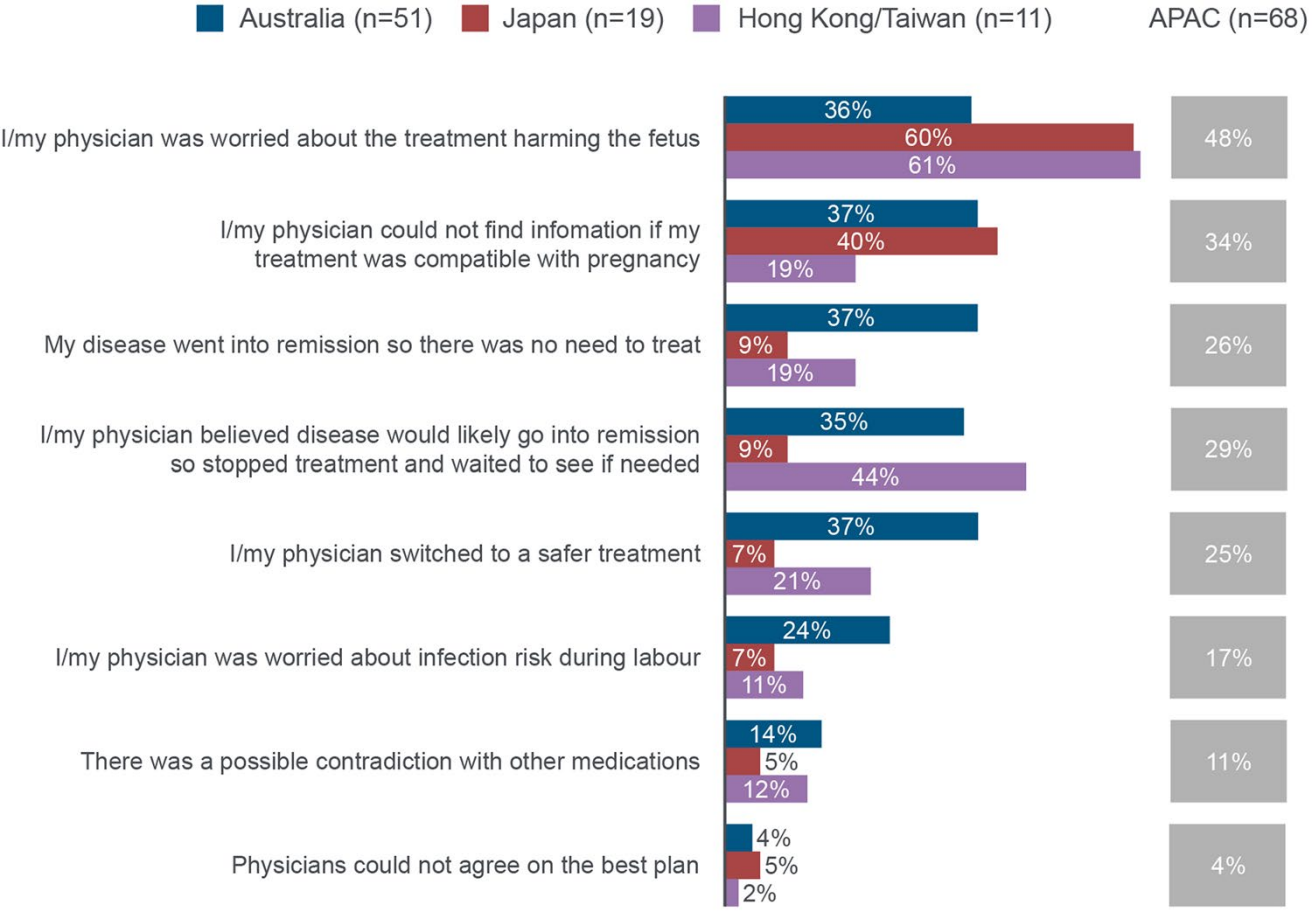
Patient-reported reasons for stopping treatment during pregnancy. ‘What were the key reasons why your treatment was stopped during your most recent pregnancy?’ The patients had stopped any of the following treatments: nonsteroidal anti-inflammatory drugs, topical products, non-biologic disease-modifying antirheumatic drugs excluding methotrexate, non-biologic disease-modifying antirheumatic drugs, methotrexate, steroids, tumour necrosis factor inhibitors, other biologics and orals. Multiple answers were possible. Due to different treatment categories having a different number of respondents, the patient numbers reported are for the drug category with the highest number of respondents. APAC: Asia–Pacific (Australia, Japan and Hong Kong/Taiwan)

Most patients (94%; *n* = 193/205) stopped their treatments for CRD around pregnancy. Treatments were more commonly stopped before pregnancy (41%; *n* = 79/192) and at the start of pregnancy (47%; *n* = 90/192), compared with during (36%; *n* = 69/192) or after pregnancy (14%; *n* = 27/192). Among patients who were receiving TNFi (*n* = 78), most (87%; *n* = 68/78) stopped TNFi therapy around pregnancy; 27% (*n* = 21/78) stopped before pregnancy, 19% (*n* = 15/78) at the start of the pregnancy, 29% (*n* = 23/78) during pregnancy and 12% (*n* = 9/78) after pregnancy. It was the treating physician’s idea 69% (*n* = 47/68) of the time to stop TNFi treatment, compared with 41% (*n* = 28/68) for obstetricians/gynaecologists and 25% (*n* = 17/68) for patients. The most frequently reported reason for stopping treatment during pregnancy was the fear of the treatment harming the fetus (Fig. 3).

#### After pregnancy

Most patients (91%; *n* = 191/210) discussed the possibility of breastfeeding with an HCP. Over half of the patients (58%; *n* = 110/210) felt they had to make a choice between treatment and breastfeeding. More patients in Japan and Hong Kong/Taiwan felt this way than those in Australia (66% [*n* = 44/67] and 79% [*n* = 27/34] versus 43% [*n* = 39/90]).

Overall, 40% (*n* = 84/210) of the patients did not breastfeed their baby. More women in Australia did not do so (58%; *n* = 63/108) compared with Japan (19%; *n* = 13/68) or Hong Kong/Taiwan (24%; *n* = 8/34). The main reason for not breastfeeding was the belief that it was unsafe when on treatment (46%; *n* = 39/84) (Supplementary Fig. 2). In 24% (*n* = 20/84) of cases, HCPs had recommended that the mother not breastfeed.

### Clinician survey

#### Participant demographics

A total of 335 clinicians met the inclusion and exclusion criteria and completed the survey. Among them, 60 (18%) were from Australia, 224 (67%) from Japan and 51 (15%) from Hong Kong/Taiwan (Supplementary Table 3). Overall, 52% of the participants were rheumatologists (*n* = 174), 25% were obstetricians (*n* = 84) and 23% were orthopaedic surgeons (*n* = 77; from Japan only). On average, 29% of female patients managed by rheumatologists and obstetricians in APAC were prescribed TNFi; orthopaedic surgeons managed the fewest women who were prescribed TNFi (19%).

#### Overall information gaps

The availability of more TNFi safety data during pregnancy was considered the most important factor for increasing clinician comfort with TNFi (rheumatologists: 86% [*n* = 150/174], obstetricians: 90% [*n* = 76/84] and orthopaedic surgeons: 75% [*n* = 58/77]; Supplementary Fig. 3).

#### Use of TNFi

**Fig. 4:**
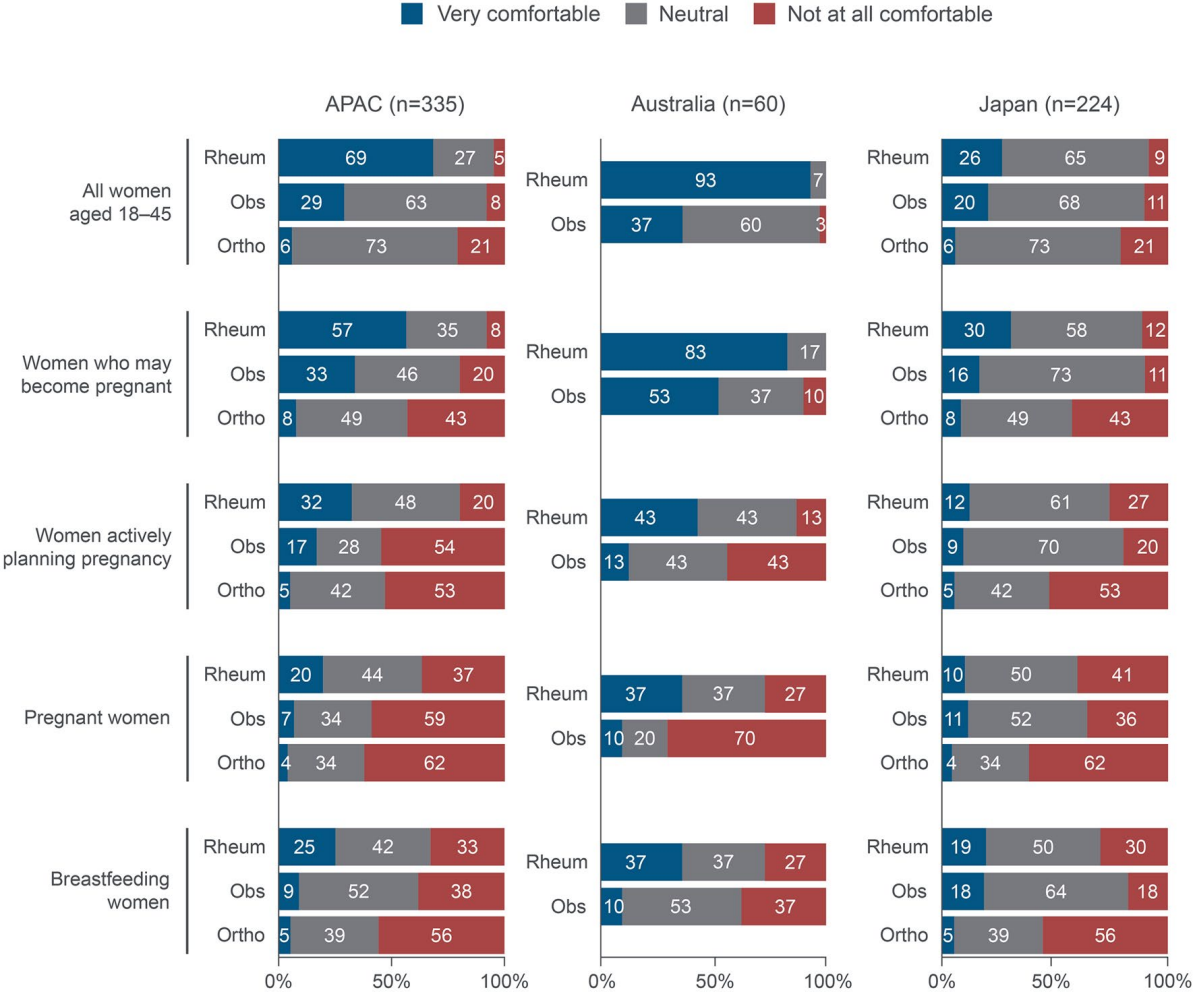
Clinicians’ level of comfort with TNFi treatment in women aged 18–45 years with CRD. ‘How comfortable are you in prescribing TNFi therapy for the following types of patients?’ APAC: *n* = 335 (rheumatologists: *n* = 174, obstetricians: *n* = 84 and orthopaedic surgeons: *n* = 77). Orthopaedic surgeons were included for Japan only. Breakdown by specialities not available for Hong Kong/Taiwan. APAC: Asia–Pacific (Australia, Japan, Hong Kong/Taiwan); CRD: chronic rheumatic disease; Obs: obstetricians; Ortho: orthopaedic surgeons; Rheum: rheumatologists; TNFi: tumour necrosis factor inhibitors

Overall, 69% (*n* = 120/174) of rheumatologists were ‘very comfortable’ for TNFi to be prescribed for female patients aged between 18 and 45 years, compared with 29% (*n* = 24/84) of obstetricians and 6% (*n* = 5/77) of orthopaedic surgeons (Fig. 4). Across specialities, comfort levels with prescribing TNFi generally decreased with the onset of family planning (32% [*n* = 56/174] of rheumatologists, 17% [*n* = 14/84] of obstetricians and 5% [*n* = 4/77] of orthopaedic surgeons). For both Australia and Japan, the comfort level of rheumatologists was generally higher than obstetricians and orthopaedic surgeons, in all groups of patients.

**Fig. 5:**
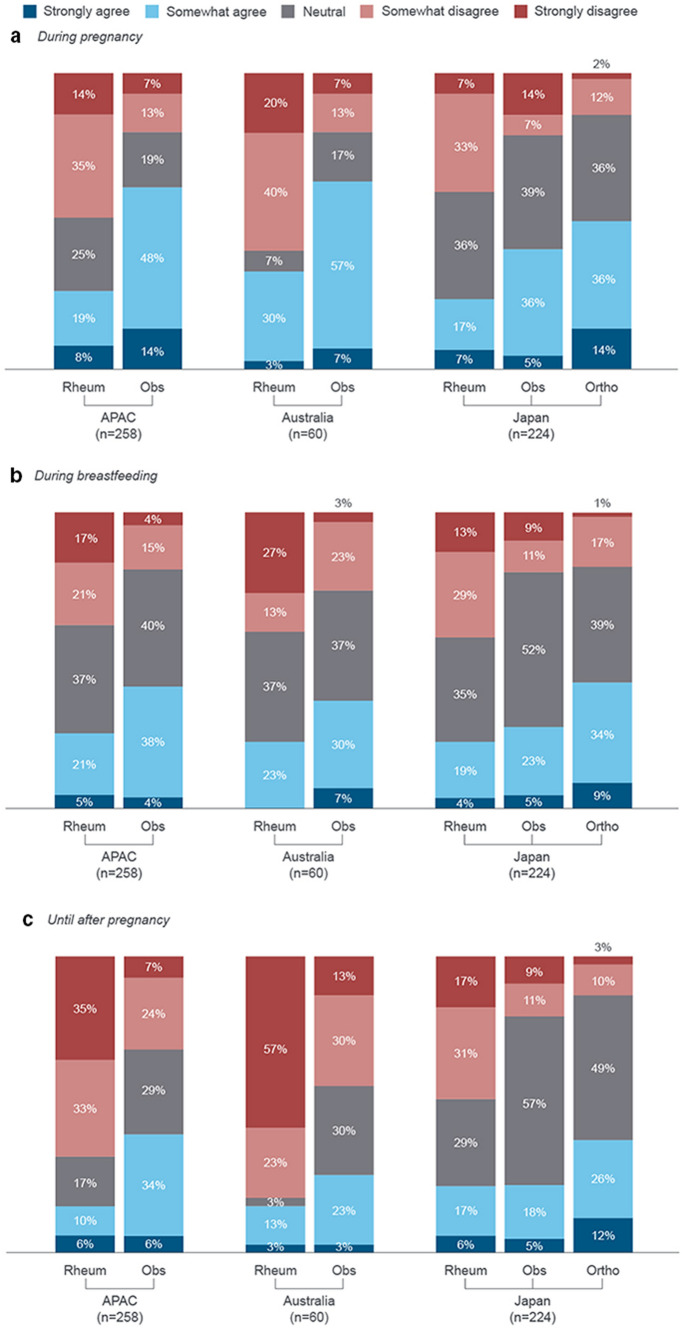
Clinicians’ level of agreement to discontinuing/avoiding TNFi treatments during pregnancy and breastfeeding. (a) ‘Once a woman becomes pregnant, she should discontinue TNFi treatment’ (b) ‘Women that are breastfeeding should not be on an TNFi agent’ (c) ‘Female patients aged 18 − 45 years should avoid TNFi therapies until after pregnancy’. APAC: *n* = 258 (rheumatologists: *n* = 174, obstetricians: n = 84). Orthopaedic surgeons were included for Japan only. Breakdown by specialities not available for Hong Kong/Taiwan. APAC: Asia–Pacific (Australia, Japan, Hong Kong/Taiwan); Obs: obstetricians; Ortho: orthopaedic surgeons; Rheum: rheumatologists; TNFi: tumour necrosis factor inhibitors

Many clinicians (43% [*n* = 75/174] of rheumatologists, 38% [*n* = 32/84] of obstetricians and 55% [*n* = 42/77] of orthopaedic surgeons) would discontinue TNFi treatment in at least 75% of female patients when they become pregnant. Obstetricians and orthopaedic surgeons were more likely than rheumatologists to recommend discontinuation of TNFi during pregnancy (62% [*n* = 52/84] and 50% [*n* = 39/77] versus 27% [*n* = 47/174]). This trend was observed both in Australia and Japan. Across specialities, more clinicians in Australia tended to recommend discontinuation of TNFi during pregnancy (Fig. 5a).

More obstetricians and orthopaedic surgeons versus rheumatologists agreed that women who are breastfeeding should not be on TNFi therapies (42% [*n* = 35/84] and 43% [*n* = 33/77] versus 26% [*n* = 45/174], Fig. 5b). More obstetricians and orthopaedic surgeons also agreed that female patients aged 18–‍45 years should avoid TNFi therapies until after pregnancy, compared with rheumatologists (40% [*n* = 34/84] and 38% [*n* = 29/77] versus 16% [*n* = 28/174], Fig. 5c). This trend was observed across Australia and Japan.

#### Disease control

**Fig. 6:**
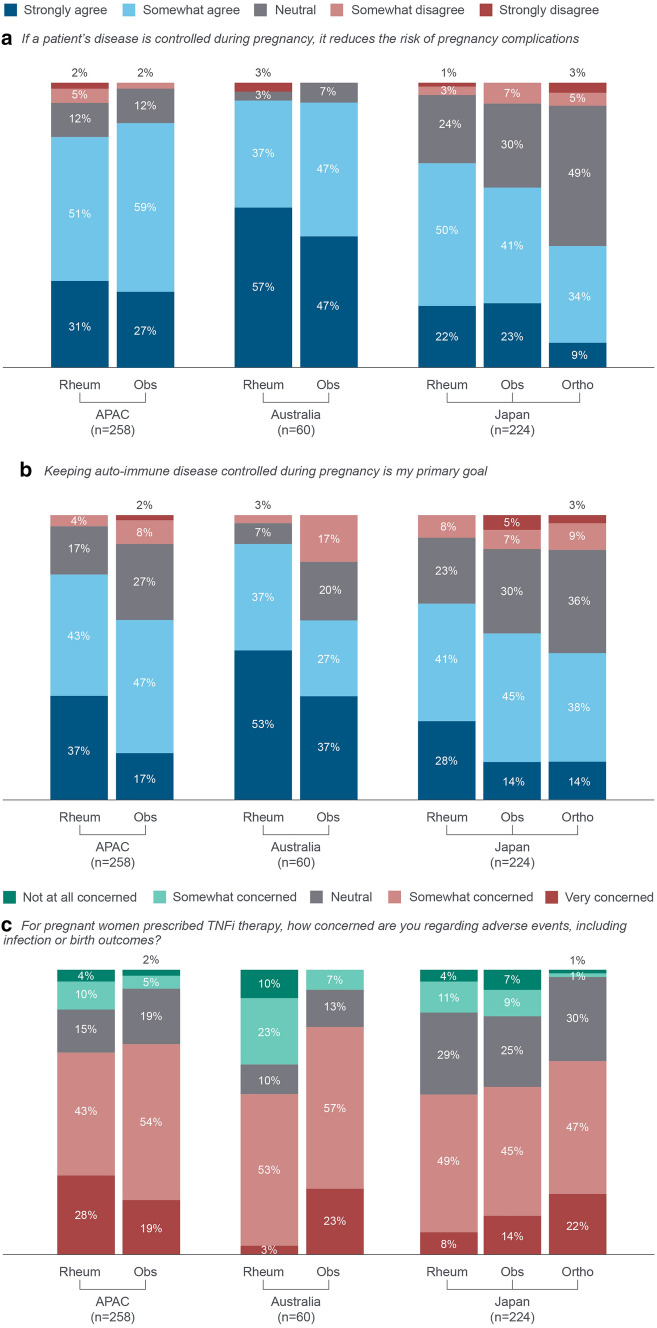
Clinicians’ attitude regarding disease control and their level of concern about AEs when TNFi treatment is prescribed during pregnancy. ‘How strongly do you agree with the following statements?’ APAC: *n* = 258 (rheumatologists: *n* = 174, obstetricians: *n* = 84). Orthopaedic surgeons were included for Japan only. Breakdown by specialities not available for Hong Kong/Taiwan. AE: adverse events; APAC: Asia–Pacific (Australia, Japan, Hong Kong/Taiwan); Obs: obstetricians; Ortho: 71 (S10) orthopaedic surgeons; Rheum: rheumatologists; TNFi: tumour necrosis factor inhibitors

Most rheumatologists (82%; *n* = 143/174) and obstetricians (86%; *n* = 72/84) agreed that controlling disease during pregnancy reduces the risk of pregnancy complications (Fig. 6a). Most clinicians in Australia (94%) agreed with this, compared with 43–72% of clinicians in Japan. More clinicians in Japan tended to have a neutral attitude towards this statement compared to those in Australia (24–‍49% versus 3–7%).

Most clinicians (rheumatologists [80%; *n* = 139/174], obstetricians [64%; *n* = 54/84] and orthopaedic surgeons [52%; *n* = 40/77]) agreed that keeping patients’ auto-immune disease controlled during pregnancy was their primary goal (Fig. 6b).

Around 70% of clinicians were concerned about AEs (including infection and birth outcomes) in women prescribed with TNFi treatment during pregnancy. Australian obstetricians and Japanese orthopaedic surgeons were the most concerned (Fig. 6c).

## Discussion

This study was conducted to explore the perspectives of women aged 18–45 years with CRD around disease management, family planning and whether they received support from their clinicians. The results demonstrate that most women felt that they did not receive all the information they needed to make informed decisions around disease management and pregnancy planning, and lacked an aligned treatment plan between HCPs involved in their care. These women also reported fears regarding pregnancy, such as passing the disease to their child, and misconceptions around treatment and breastfeeding, feeling that they had to choose between breastfeeding and treatment; this was similar across the APAC countries surveyed. Additionally, a separate survey was conducted to understand clinicians’ attitudes towards the use of TNFi in women 18–‍45 years of age with CRD. The study results found that while most clinicians agreed that controlled disease reduced the risk of pregnancy complications, control of disease activity is not yet consistently implemented. Many clinicians were uncertain about the appropriate treatment to use during pregnancy. The results of these surveys highlight a need for improved education and dissemination of information on the management of CRD in women aged 18–‍45 years, for both patients and HCPs.

Disease control is important in the management of CRD in women as active disease may lead to complications during pregnancy and adverse birth outcomes [6, 15, 16]. In this study, the importance of disease control was recognised by many of the APAC clinicians. However, there was still a proportion who were neutral or in disagreement that if a patient’s disease is controlled during pregnancy, it reduces the risk of pregnancy complications, and who did not prioritise disease control during pregnancy. This could be attributed to a lack of understanding of the potential risks associated with discontinuing treatment during pregnancy. Prescribing habits could also be impacted by the lack of experience among clinicians managing CRD in female patients who become pregnant [12]. In a recent study in the U.S. and Europe, 41% of physicians reported having suboptimal skills in adjusting treatment according to the different life stages of their female patients [17]. Similarly, many clinicians in this APAC survey were uncertain about the appropriate treatment to use during pregnancy. Although there are recommendations on the management of CRD in women, clinicians may not be familiar with these due to factors such as language barriers and lack of active dissemination [12]. In addition, clinicians may not know how to apply these recommendations in clinical practice due to a lack of practical guidance and knowledge of who to consult [18]. Development of and access to living documents on medications and patient information, roundtable discussions and small conferences among physicians could be considered to keep physicians updated on improvements in the management of CRD in female patients.

Comfort level with prescribing TNFi in women aged 18–45 years with CRD was generally higher among rheumatologists than obstetricians and orthopaedic surgeons. A recent systematic review identified insufficient knowledge of treatment guidelines and fears around the safety of medication in both mother and fetus as the main factors that influence clinician behaviour in treating women with chronic inflammatory diseases [12]. Similarly, this study found the factors most likely to improve physician comfort with TNFi treatment were more safety data during pregnancy and on the child, and lactation data. In general, orthopaedic surgeons who participated in this survey were more conservative compared with rheumatologists or obstetricians. Orthopaedic surgeons in this study were less comfortable with using TNFi in women aged 18–45 years, more likely to avoid using TNFi during pregnancy and breastfeeding, and around 50% of them did not view disease control as their primary goal during pregnancy. Further studies would be required to look into the reasons behind the conservative approach that clinicians may have, and strategies to overcome them. However, since clinicians are involved in different aspects of patient care, development of practical guidance tailored to the clinician group could be useful to guide them on implementing the evidence into their routine clinical practice.

Despite over 81% of the APAC clinicians reporting that they typically co-manage the auto-immune disease with other specialities during pregnancy, many patients reported that they lacked an aligned treatment plan. According to the results of a patient survey conducted in the U.S., patients wanted their rheumatologists to communicate directly with their obstetrician/gynaecologist, rather than using the patients as an intermediary [19]. Similarly, the results of the APAC survey reported here highlight the need for a clear management plan of shared care and early involvement of multiple stakeholders in all women aged 18–45 years. Communication and exchange of information between the treating physician and the obstetrician/gynaecologist should thus be part of routine care, as patients may consult them independently.

In this study, one in two women in APAC had unaddressed concerns that delayed their decision to get pregnant, the most common being passing on a health issue to their baby. Most women in APAC did not feel fully supported or informed during pregnancy planning and only one-third of them felt they had received all the information they needed during pregnancy. In a survey conducted in the U.S. in women aged 18–45 years with CRD, patients strongly expressed their wish for their rheumatologists to play an active role in their family planning care. The women wanted their rheumatologists to initiate conversations about family planning continuously to provide clear and comprehensive information about their disease and treatments around pregnancy [19]. From interviews conducted with patients with RA in Australia, although most patients depended on their rheumatologist as their primary source of information, there was still an unmet demand for more information, especially around the safety of medication during pregnancy and breastfeeding [20]. This may also be the expectations of the women who participated in this survey. As such, education of clinicians is important for them to initiate and engage in discussions with their patients and to provide consistent, accurate and clear information to patients. The EULAR recommendations for the management of pregnant and lactating patients with rheumatic disease suggested that family planning and adjustment of treatment should be addressed in all patients of reproductive age [3]. A clinical panel in Australia developed a set of clinician-centred, practical suggestions to complement the EULAR recommendations to aid information delivery and clinical practices to support women with RA [18]. The American College of Rheumatology (ACR) has also recently published recommendations on the management of patients with CRD throughout their reproductive lifespan, complementing the current recommendations which focused more on the management of patients who are pregnant and lactating [10]. In previous studies, patients identified that joining a community of people with similar experiences was helpful for social support and sharing their concerns and anxieties around family planning [20, 21]. Given this, clinicians can advise on the availability of patient support groups in their respective regions, for patients to receive additional support in their family planning journey.

The recent ACR recommendations on management of reproductive health in patients with rheumatic diseases suggested many medications, such as hydroxychloroquine, sulfasalazine, rituximab and all TNFi, may be used during lactation [10]. In a prospective cohort study conducted in 2019 in the Netherlands, women with RA were reported to be less likely to breastfeed their baby as compared with the general women population. In the same study, it was reported that the main reason for discontinuation of breastfeeding was the restarting of medication (57.8%, 129/223), although in around 40% of the patients (41.1%, 53/129), their medications were considered compatible with breastfeeding [22]. In this APAC study, more than half of the women felt they had to choose between breastfeeding and treatment. In this survey, more women in Australia did not breastfeed their baby as compared with Japan or Hong Kong/Taiwan, and this was similar to the general population where there was a higher proportion of children who were exclusively breastfed at 6 months in Japan (34.7%) than in Australia (14.0%) [23]. Despite this, the main reason for not breastfeeding was the same across all APAC countries, i.e., a lack of information and fear that the treatment might harm their baby. These results suggest that all patients should be informed about the risk versus benefit of disease control during breastfeeding and all compatible treatment options.

The limitations of these surveys include a lack of formal validation for the questionnaires, a small number of participants, and self-reporting of disease severity by the participants. This study included only women who had successful pregnancies and their experience may be different from those who were unsuccessful. However, this was because this study aimed to understand the women’s experience throughout the whole pregnancy journey, from planning a family, to being pregnant and giving birth. As most respondents were from Australia and Japan, the findings may not be generalisable across the whole of APAC due to diversity in ethnicities and cultural differences. Differences in the responses received may also be attributed to the different social cultures of each country; however, this study was not designed to compare differences between countries. As with epidemiological studies, there may be recall bias as the patients had to recall events from when they were pregnant in the past 2–‍5 years. Patients who had other CRD, such as systemic lupus erythematosus, and clinicians who were managing these patients were not included in this study. These patients and clinicians may face similar challenges, and comparison between the different populations would be helpful to determine if the fears and misconceptions are specific to rheumatic diseases. Questions in the physicians’ survey were also tailored towards TNFi; it may be helpful if attitudes and perceptions around the use of other classes of treatments were assessed. Lastly, as with all surveys, it is important to note that the results may not be truly reflective of the general CRD population, as the sample who participated may have stronger opinions compared with those who did not respond.

## Conclusion

In APAC, fears and misconceptions are common among women with CRD. They often had unaddressed concerns and did not feel fully informed in their family planning decisions. Despite many clinicians agreeing that controlled disease reduces the risk of pregnancy complications, control of disease activity was not yet consistently implemented, and many clinicians were uncertain about the appropriate treatments to use during pregnancy. There is a need for patient and physician access to up-to-date and accurate information on the risk versus benefit of different treatment options for disease management in women with CRD. A plain language summary of this manuscript is provided in Supplementary Fig. 4.

## Supplementary Information

Below is the link to the electronic supplementary material.

Supplementary file1 (DOCX 293 KB)
