# Supplementary material for: Management of chronic rheumatic diseases in women 18–45 years of age in Asia Pacific: insights from patient and clinician surveys
Source: Rheumatol Int. 2022 Sep 26;43(4):721–33. doi: 10.1007/s00296-022-05206-0 (PMC9995525; doi:10.1007/s00296-022-05206-0)

*Rheumatology International*

Management of Chronic Rheumatic Diseases in Women 18–45 Years of Age in Asia Pacific: Insights from Patient and Clinician Surveys

SUPPLEMENTARY TABLES

Supplementary Table 1. Patient survey

| **Question^a^** | **Response** |
| --- | --- |
| 1. When were you diagnosed with your (insert diagnosis)? [single response] | - Before your pregnancy - During your pregnancy - After your pregnancy |
| 2. Did you ever use any medications for your (insert diagnosis)? [single response] | - Yes - No |
| 3. With which healthcare professional did you discuss at the different stages of your pregnancy? [multiple response] | \|  \| Before (when you start planning) your pregnancy \| At the start of your pregnancy \| During your pregnancy \| After your pregnancy \| In none of these situations \| \| --- \| --- \| --- \| --- \| --- \| --- \| \| Rheumatologists (show only for RA, axSpA, PsA) \|  \|  \|  \|  \|  \| \| Dermatologists (show only for PsA and PsO) \|  \|  \|  \|  \|  \| \| GP \|  \|  \|  \|  \|  \| \| OBGYN \|  \|  \|  \|  \|  \| \| Paediatrician \|  \|  \|  \|  \|  \| \| Midwife \|  \|  \|  \|  \|  \| |
| 3a. Before pregnancy, which one did you consider your most critical advisor? [single response, depending on answer in 3] | - Rheumatologists (show only for RA, axSpA, PsA) - Dermatologists (show only for PsA and PsO) - GP - OBGYN - Paediatrician - Midwife |
| 4. Prior to pregnancy, during your consultation with this healthcare professional, who brought up the subject of family planning? [single response, depending on answer in 3a] | - Myself - Physician/ Healthcare professional from question 3a above - My partner - I do not remember |
| 5. Can you still remember when the topic of planning a pregnancy was discussed for the first time with a healthcare professional? [single response] | - At the time of diagnosis - At the time of treatment initiation - At a regular visit - I do not remember |
| 6. If you think back about this discussion, to what extent did you … [select level] | \|  \| 1  not at all \| 2 \| 3 \| 4 \| 5 \| 6 \| 7 \| 8 \| 9 \| 10 com-pletely \| \| --- \| --- \| --- \| --- \| --- \| --- \| --- \| --- \| --- \| --- \| --- \| \| … feel like your questions and concerns about pregnancy were addressed \|  \|  \|  \|  \|  \|  \|  \|  \|  \|  \| \| …have the support of your healthcare professional on getting pregnant \|  \|  \|  \|  \|  \|  \|  \|  \|  \|  \| |
| 7. Did you have any concern that delayed your decision to become a mother for your most recent pregnancy? [single response] | - Yes - No |
| 8. Which (if any) of the following concerns delayed your decision to become a mother for your most recent pregnancy? [multiple response] | - I was not emotionally ready to become a parent - My partner was not emotionally ready to become a parent - I was not financially able to support a child/go on maternity leave - My relationship with my partner was not in the right place to have a child - I was not physically healthy enough to conceive and carry a child to term - I might pass on a health issue to my child - My education/career was not in the right place to have a child - I thought I was too young to have a child - I thought I was too old to have a child - I did not have the support of my physician to become pregnant - I did not have access to the medical care needed to become pregnant and give birth - Other, please specify: - None of the above |
| 9. Which of the following best describes the circumstances around your most recent pregnancy?  [single response] | - I was actively trying to get pregnant - I was open to seeing what happened (wasn’t trying particularly hard one way or the other) - I was not thinking about this - I was actively trying to NOT get pregnant |
| 10. How much time did it take you to become pregnant from the moment you started trying to conceive? | - ____ months - I don’t know |
| 11. At the time you discovered you were pregnant, to what extent did you experience any of the following concerns? [single response] | \|  \| Not at all concerning \|  \|  \|  \| Very concerning \| Not applicable \| \| --- \| --- \| --- \| --- \| --- \| --- \| --- \| \| That the pregnancy might end in miscarriage \|  \|  \|  \|  \|  \|  \| \| That I was not physically healthy enough to be pregnant \|  \|  \|  \|  \|  \|  \| \| That my disease activity would compromise the health of the baby \|  \|  \|  \|  \|  \|  \| \| That I might pass on a health issue to my child \|  \|  \|  \|  \|  \|  \| \| That there might be something wrong with the child \|  \|  \|  \|  \|  \|  \| \| That pregnancy might make my disease worse \|  \|  \|  \|  \|  \|  \| \| That I did not have access to the medical care needed to have a successful pregnancy and give birth \|  \|  \|  \|  \|  \|  \| \| That the treatment I was on might harm the baby \|  \|  \|  \|  \|  \|  \| |
| 12. Prior to pregnancy, did you receive all the information you needed from your healthcare professional? Or was there any type of information you would have liked to receive and that you did not get? [multiple response] | - I received all the information I needed (fixed position) - Impact of the disease activity on my health - Impact of my disease activity on my baby - Impact of the treatment on my baby - Impact of pregnancy on my disease - Other, please specify: |
| 13. What treatment were you taking before your pregnancy? [multiple response] | **Options for RA patients**   - Nonsteroidal anti-inflammatory drugs (e.g. diclofenac, ibuprofen) - Non-biologic disease modifying antirheumatic drug excluding MTX (e.g., sulfasalazine, hydroxychloroquine) - Methotrexate - Steroids - Tumour necrosis factor inhibitors (e.g., Enbrel or Brenzys [Australia only; etanercept], Simponi [golimumab], Humira [adalimumab], Remicade [infliximab], Cimzia [certolizumab pegol]) - Other biologic (e.g., Actemra/RoActemra [tocilizumab], Rituxan [rituximab]) - Other oral (e.g., Xeljanz [tofacitinib], Olumiant [baracitib]) - I don’t know   **Options for axSpA patients**   - Nonsteroidal anti-inflammatory drugs (e.g., diclofenac, ibuprofen) - Non-biologic disease modifying antirheumatic drug (e.g., sulfasalazine, hydroxychloroquine) - Steroids - Tumour necrosis factor inhibitors (e.g., Enbrel or Brenzys [Australia only; etanercept], Simponi [golimumab], Humira [adalimumab], Remicade [infliximab], Cimzia [certolizumab pegol]) - Other biologic (including Cosentyx [secukinumab], Taltz [ixekizumab]) - I don’t know   **Options for PsA patients**   - Nonsteroidal anti-inflammatory drugs (e.g., diclofenac, ibuprofen) - Topical products (e.g., calciprotriol, calcitriol, clobetasol, calcipetril/betamethasone) - Non-biologic disease modifying antirheumatic drug excluding MTX (e.g., sulfasalazine, hydroxychloroquine) - Methotrexate - Steroids - Tumour necrosis factor inhibitor (e.g., Enbrel or Brenzys [Australia only; etanercept], Simponi [golimumab], Humira [adalimumab], Remicade [infliximab], Cimzia [certolizumab pegol] - Other biologic (including Stelara [ustekinumab], Cosentyx [secukinumab], Taltz [ixekizumab]) - Other oral (e.g., Otezla [apremilast]) - I don’t know   **Options for PsO patients**   - Immunosuppressants (e.g., ciclosporine) - Topical products (e.g., calciprotriol, calcitriol, clobetasol, calcipetril/betamethasone) - Tumour necrosis factor inhibitor (e.g., Enbrel [etanercept], Humira [adalimumab], Remicade [infliximab]) - Other biologic (including Stelara [ustekinumab], Cosentyx [secukinumab], Taltz [ixekizumab]) - Other oral (e.g., Otezla [apremilast]) - I don’t know |
| 14. Before trying to conceive, did you and your treating physician create a treatment plan around what medications you would take and not take if you became pregnant? [single response] | - Yes - No |
| 15. Was there any discussion around your treatment plan between your treating physician and your obstetrician/gynaecologist? [single response] | - Yes - No |
| 16. Did you stop any of the following treatment and when? [single response] | \|  \| Before (when you start planning) your pregnancy \| At the start of your pregnancy \| During your pregnancy \| After your pregnancy \| In none of these situations \| \| --- \| --- \| --- \| --- \| --- \| --- \| \| Same list as question 13 \|  \|  \|  \|  \|  \| \|  \|  \|  \|  \|  \|  \| \|  \|  \|  \|  \|  \|  \| |
| 17. You indicated you stopped your treatment. Whose idea was it to stop your treatment?  [multiple response, based on Q3 and Q16 response] | - My idea - My treating physician idea - My obstetrician/gynaecologist idea - My partner’s idea - Other, please specify: |
| 18. What were the key reasons why your treatment was stopped during your most recent pregnancy?  [multiple response, based on Q16] | - I/my physician [based on answer in Q17] was worried about the treatment harming the fetus - I/my physician [based on answer in Q17] could not find information if my treatment was compatible with pregnancy - My disease went into remission, so there was no need to treat - I/my physician [based on answer in Q17] believed disease would likely go into remission, so stopped treatment and waited to see if needed - I switched to a safer treatment/my physician switched me to a safer treatment [based on answer in Q17] - I/my physician [based on answer in Q17] worried about infection risk during labour - There was a possible contraindication with other medications - Physicians could not agree on the best plan - Other |
| 19. How would you rate the severity of your (insert diagnosis) during your pregnancy? [single response] | - Very well controlled/in remission - Well controlled/in low disease activity - Not very well controlled/in medium disease activity - Uncontrolled/in high disease activity |
| 20. Did your disease improve, worsen or stay the same during your pregnancy? [single response] | - Improved a lot - Improved a little - Stayed the same - Worsened a little - Worsened a lot |
| 21. After your baby was born, did your disease improve, worsen or stay the same? [single response] | - Improved a lot - Improved a little - Stayed the same - Worsened a little - Worsened a lot |
| 22. How many months after your baby was born did it worsen? | - ____ months after I gave birth |
| 23. You indicated that you stopped your treatment before or during pregnancy. Did you reinitiate after your pregnancy? If so, how effective was the treatment when reinitiated? [single response, based on Q16] | - I did not reinitiate - I did reinitiate and it was more effective - I did reinitiate and it was less effective - I did reinitiate and it was as effective as before - I don’t remember |
| 24. Did you breast-feed your baby? [single response] | - Yes - No |
| 25. You indicated that you did not breast-feed your baby. Can you indicate what the reason you had for not doing so? [single response, based on Q24] | - Needed to start treatment for my illness immediately and could not breastfeed while on treatment - Needed to go back to work - Did not want to breastfeed - Was physically unable to breastfeed - Baby did not latch - Doctor/healthcare professional recommended not to breastfeed - Other, please specify: - None of the above |
| 26. Did your treating physician talk to you about the possibilities of breastfeeding? [single response] | - Yes, we talked about this for the first time before my pregnancy - Yes, we talked about this for the first time during my pregnancy - Yes, we talked about this for the first time right after delivery - No, this option was not discussed |
| 27. Which other healthcare professional(s) discussed the possibilities of breastfeeding with you? [multiple response] | - Primary Care Physician/General Practitioner - Obstetrician or Gynaecologist - Paediatrician - Lactation Specialist - Midwife - Other, please specify: - I didn’t consult a healthcare professional at this point |
| 28. Can you also indicate which of the healthcare professionals was the most important in influencing your decision whether or not to breastfeed?  [single response, based on Q26 and Q27 response] | - Primary Care Physician/General Practitioner - Rheumatologist (option for RA/axSpA/PsA patients only) - Dermatologist (option for PsO/PsA patients only) - Obstetrician or Gynaecologist - Paediatrician - Lactation Specialist - Midwife - Other, please specify: |
| 29. To what extent did you and/or your healthcare professionals worry about the consequences of your treatment on your baby while breastfeeding?  [multiple response, based on Q23 response] | \|  \| 1 not at all concerned \|  \|  \|  \|  \|  \|  \|  \|  \| 10 very concerned \| \| --- \| --- \| --- \| --- \| --- \| --- \| --- \| --- \| --- \| --- \| --- \| \| Myself \|  \|  \|  \|  \|  \|  \|  \|  \|  \|  \| \| My gynaecologist or paediatrician or lactation specialist \|  \|  \|  \|  \|  \|  \|  \|  \|  \|  \| \| My Rheumatologist (option for RA/axSpA/PsA patients only)/Dermatologist (option for PsO/PsA patients only) \|  \|  \|  \|  \|  \|  \|  \|  \|  \|  \| |
| 30. Did you feel you had to make a choice between breastfeeding and treatment? [multiple response] | - Yes, I had the feeling that I could not combine both breastfeeding and my treatment - No, I had the feeling that I could combine both breastfeeding and my treatment |
| 31. How reliable do you feel the information from your healthcare professional(s) is on how to manage your disease during pregnancy? [single response per row] | \|  \| 1 Not at all reliable \| 2 \| 3 Neither reliable, nor unreliable \| 4 \| 5 Very reliable \| Not applicable for me \| \| --- \| --- \| --- \| --- \| --- \| --- \| --- \| \| Primary Care Physician/General Practitioner \|  \|  \|  \|  \|  \|  \| \| Rheumatologist (option for RA/axSpA/PsA patients only) \|  \|  \|  \|  \|  \|  \| \| Midwife \|  \|  \|  \|  \|  \|  \| \| Dermatologist (option for PsO/PsA patients only) \|  \|  \|  \|  \|  \|  \| \| Obstetrician or Gynaecologist \|  \|  \|  \|  \|  \|  \| \| Paediatrician \|  \|  \|  \|  \|  \|  \| \| Other; please specify […] \|  \|  \|  \|  \|  \|  \| |
| 32. How consistent do you feel the information you received from different healthcare professionals is regarding the following? [single response per row] | \|  \| 1 Very conflicting \| 2 \| 3 Neither conflicting, nor consistent \| 4 \| 5 Very consistent \| I did not receive any information on this topic \| \| --- \| --- \| --- \| --- \| --- \| --- \| --- \| \| Medications to take during pregnancy \|  \|  \|  \|  \|  \|  \| \| What to do prior to becoming pregnant \|  \|  \|  \|  \|  \|  \| \| How to manage pregnancy overall \|  \|  \|  \|  \|  \|  \| \| When to stop medications \|  \|  \|  \|  \|  \|  \| \| Risk of medication on your pregnancy outcome \|  \|  \|  \|  \|  \|  \| \| Medications to take during breastfeeding \|  \|  \|  \|  \|  \|  \| |

^a^Screening and demographic questions are not included.

Supplementary Table 2. Clinician surveys

| **Question^a^** | **Response** |
| --- | --- |
| Q1. What percentage of your female patients with autoimmune disorders fall into the following age groups? [numeric response; sum up to 100%] | - <18 years old - 18–35 - 36–45 - 46–64 - 65+ |
| Q2. What percentage of your female patients between the age of 18–45 with autoimmune disorders (Rheumatoid Arthritis, Psoriatic Arthritis and Axial Spondyloarthritis) do you currently prescribe a TNFi treatment? [numeric response; range 0–100%] | - Please enter a number |
| Q3. How comfortable are you in prescribing TNFi therapy for the following types of patients? [single response per row] | \|  \| Not at all comfortable \| Neutral \| Very comfortable \| \| --- \| --- \| --- \| --- \| \| All female patients between the age of 18–45 \| 1 \| 2 \| 3 \| \| Female patients who may become pregnant within the next few years \| 1 \| 2 \| 3 \| \| Female patients who are actively trying to become pregnant \| 1 \| 2 \| 3 \| \| Female patients who are pregnant \| 1 \| 2 \| 3 \| \| Female patients who are breast feeding \| 1 \| 2 \| 3 \| |
| Q4. Do you recommend that your female patients discontinue a TNFi agent prior to pregnancy? [single response] | - Yes - No |
| Q5. How many months prior to pregnancy would you recommend that TNFi therapy is discontinued? [single response, based on Q4 response] | - Immediately prior / as soon as pregnancy is suspected and/or confirmed - Up to 3 months prior - 3 to less than 6 months prior - 6 to less than 9 months prior - 9 to less than 12 months prior - 12 months+ prior |
| Q6. For your female patients who become pregnant while they are taking a TNFi agent, in what percentage of patients is the decision made to discontinue her biologic treatment? [single response] | - For less than 10% of patients - For 10% to less than 25% of patients - For 25% to less than 50% of patients - For 50% to less than 75% of patients - For 75% or more of patients |
| Q7. Hypothetically, what, if anything, would make you more comfortable with using TNFi agents among female patients between the age of 18-45 who may become pregnant in the future? [multiple response] | - More safety data for child 5 years post delivery - More safety data during pregnancy - More lactation data - A multidisciplinary discussion (Obstetrician etc). - Recommendation from a Scientific Leader (Key Opinion Leader) - Nothing |
| Q8. How strongly do you agree with the following statements? [single response per row] | \|  \| Strongly Disagree \| Somewhat Disagree \| Neutral \| Somewhat Agree \| Strongly Agree \| \| --- \| --- \| --- \| --- \| --- \| --- \| \| Female patients 18-45 should avoid TNFi therapies until after pregnancy \| 1 \| 2 \| 3 \| 4 \| 5 \| \| Once a woman becomes pregnant she should discontinue TNFi treatment \| 1 \| 2 \| 3 \| 4 \| 5 \| \| Women that are breastfeeding should not be on a TNFi agent \| 1 \| 2 \| 3 \| 4 \| 5 \| \| If a patient’s disease is controlled during pregnancy, it reduces the risk of pregnancy complications \| 1 \| 2 \| 3 \| 4 \| 5 \| \| Keeping auto-immune disease controlled during pregnancy is my primary goal \| 1 \| 2 \| 3 \| 4 \| 5 \| \| During pregnancy I typically co-manage the auto-immune disease with other specialities (e.g. Obstetricians and Gynaecologists, Primary Care Physicians etc.) \| 1 \| 2 \| 3 \| 4 \| 5 \| \| If there was a TNFi that could be used throughout pregnancy, I would be comfortable that my patients stay under treatment with this TNFi throughout their pregnancy \| 1 \| 2 \| 3 \| 4 \| 5 \| |
| Q9. For women who are pregnant and are prescribed TNFi therapy, how concerned are you regarding adverse events, including infection or birth outcomes? [single response] | - Very concerned - Somewhat concerned - Neutral - Somewhat unconcerned - Not at all concerned |
| [Additional question for Obstetricians only]  How often do you contact the prescriber or have a multidisciplinary discussion with the patient’s other physicians prior to change or discontinuation of TNFi treatment during pregnancy?  [single response] | - For less than 10% of patients - For 10% to less than 25% of patients - For 25% to less than 50% of patients - For 50% to less than 75% of patients - For 75% or more of patients |

^a^Screening and demographic questions are not included.

Supplementary Table 3. Participant demographics

| **Patients** | **APAC (n=210)** | **Australia (n=108)** | **Japan (n=68)** | **Hong Kong/ Taiwan**  **(n=34)** |
| --- | --- | --- | --- | --- |
| **Age group, years, n (%)** |  |  |  |  |
| 18–30 | 51 (24) | 31 (29) | 19 (28) | 1 (3) |
| 31–40 | 133 (63) | 67 (62) | 35 (51) | 31 (91) |
| 41–45 | 26 (12) | 10 (9) | 14 (21) | 2 (6) |
| **Pregnant, n (%)** |  |  |  |  |
| In the past 2 years | 113 (54) | 56 (52) | 38 (56) | 19 (56) |
| 2–5 years ago | 97 (46) | 52 (48) | 30 (44) | 15 (44) |
| **Disease indication, n (%)** |  |  |  |  |
| RA | 122 (58) | 51 (47) | 51 (75) | 20 (59) |
| PsA | 48 (23) | 27 (25) | 17 (25) | 4 (12) |
| axSpA | 40 (19) | 30 (28) | 0 (0) | 10 (29) |
| **Self-reported disease severity, n (%)** |  |  |  |  |
| Moderate | 162 (77) | 68 (63) | 61 (90) | 33 (97) |
| Severe | 48 (23) | 40 (37) | 7 (10) | 1 (3) |
| **Time of diagnosis of CRD, n (%)** |  |  |  |  |
| Before pregnancy | 151 (72) | 73 (68) | 55 (81) | 23 (68) |
| During pregnancy | 59 (28) | 35 (32) | 13 (19) | 11 (32) |
| **Medications taken before pregnancy, n (%)** |  |  |  |  |
| TNFi biologics | 78 (37) | 57 (53) | 14 (21) | 7 (21) |
| Other biologics | 27 (13) | 13 (12) | 13 (19) | 1 (3) |
|  |  |  |  |  |
| **Clinicians** | **APAC (n=335)** | **Australia (n=60)** | **Japan (n=224)** | **Hong Kong/ Taiwan**  **(n=51)** |
| **Specialisation,^a^ n (%)** |  |  |  |  |
| Rheumatologists | 174 (52) | 30 (50) | 103 (46) | 41 (80) |
| Obstetricians | 84 (25) | 30 (50) | 44 (20) | 10 (20) |
| Orthopaedic surgeons^b^ | 77 (23) | N/A | 77 (34) | N/A |
| **Proportion of women aged 18–‍45 years prescribed TNFi treatment, mean (SD)** |  |  |  |  |
| Rheumatologists | 29 (19.4) | 34 (18.8) | 26 (21.3) | N/A |
| Obstetricians | 29 (22.9) | 28 (23.8) | 34 (27.4) | N/A |
| Orthopaedic surgeons^a^ | 19 (24.0) | N/A | 19 (24.0) | N/A |

^a^Breakdown by specialities not available for Hong Kong/Taiwan. ^b^Orthopaedic surgeons were included for Japan only. APAC: Asia-Pacific (Australia, Japan, Hong Kong/Taiwan); axSpA: axial spondyloarthritis; CRD: chronic rheumatic disease; N/A: not available; PsA: psoriatic arthritis; RA: rheumatoid arthritis; SD: standard deviation; TNFi: tumour necrosis factor inhibitors.

SUPPLEMENTARY FIGURES

Supplementary Figure 1. Sufficiency of information provided by HCPs


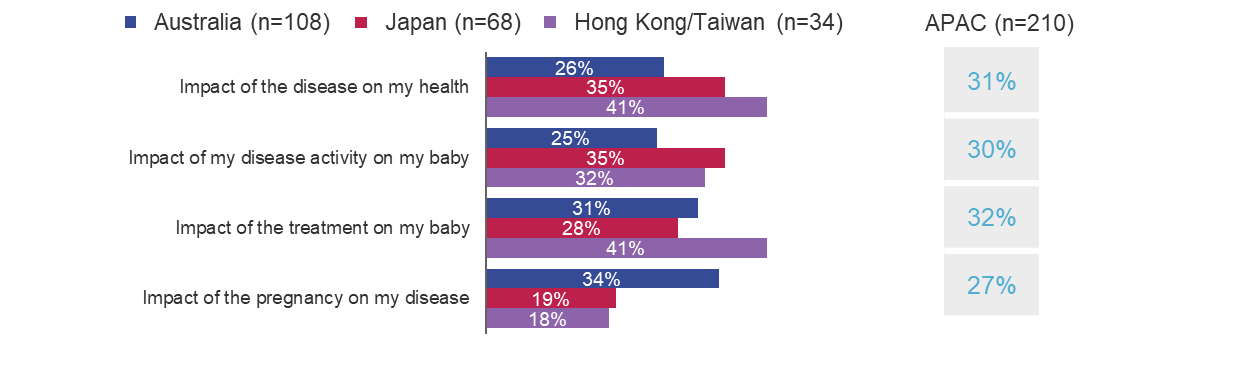


‘Prior to the pregnancy did you receive all the information you needed from your HCP?’ Multiple answers were possible. APAC: Asia-Pacific (Australia, Japan and Hong Kong/Taiwan); HCP: Healthcare professional.

Supplementary Figure 2. Patient-reported reasons for not breastfeeding


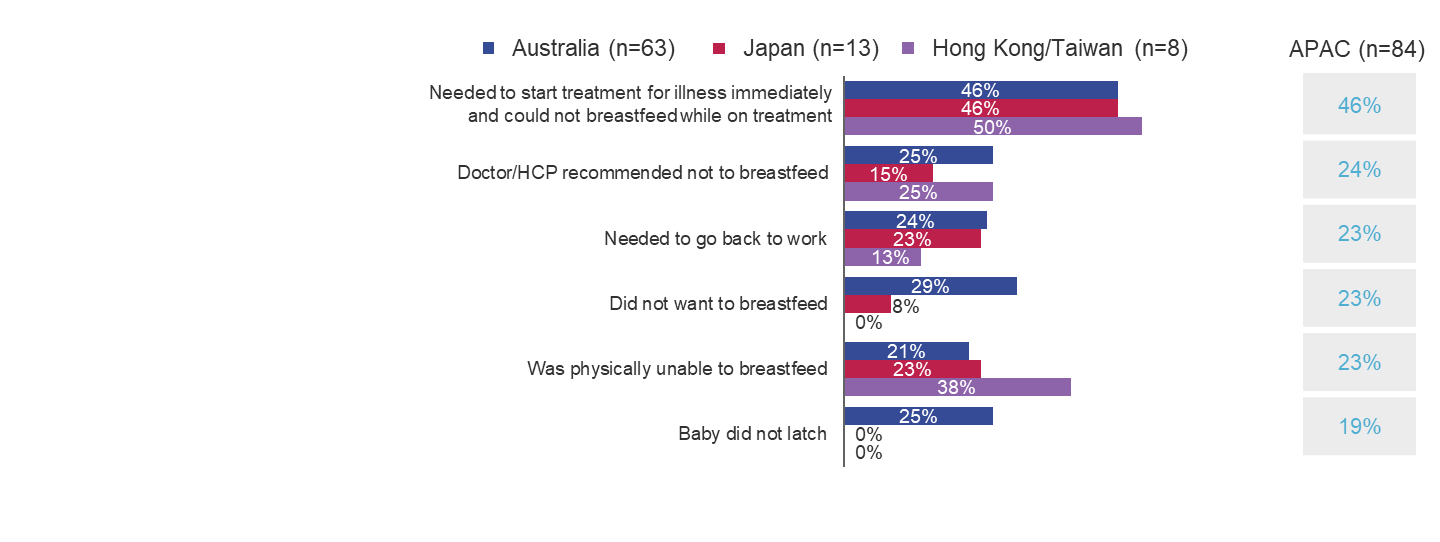


‘You indicated that you did not breastfeed your baby. Can you indicate what reasons you had for not doing so?’ Due to the low number of patients, these data should be interpreted with caution. Multiple answers were possible. APAC: Asia-Pacific (Australia, Japan and Hong Kong/Taiwan); HCP: healthcare professional.

Supplementary Figure 3. Factors increasing clinicians’ comfort with TNFi treatment


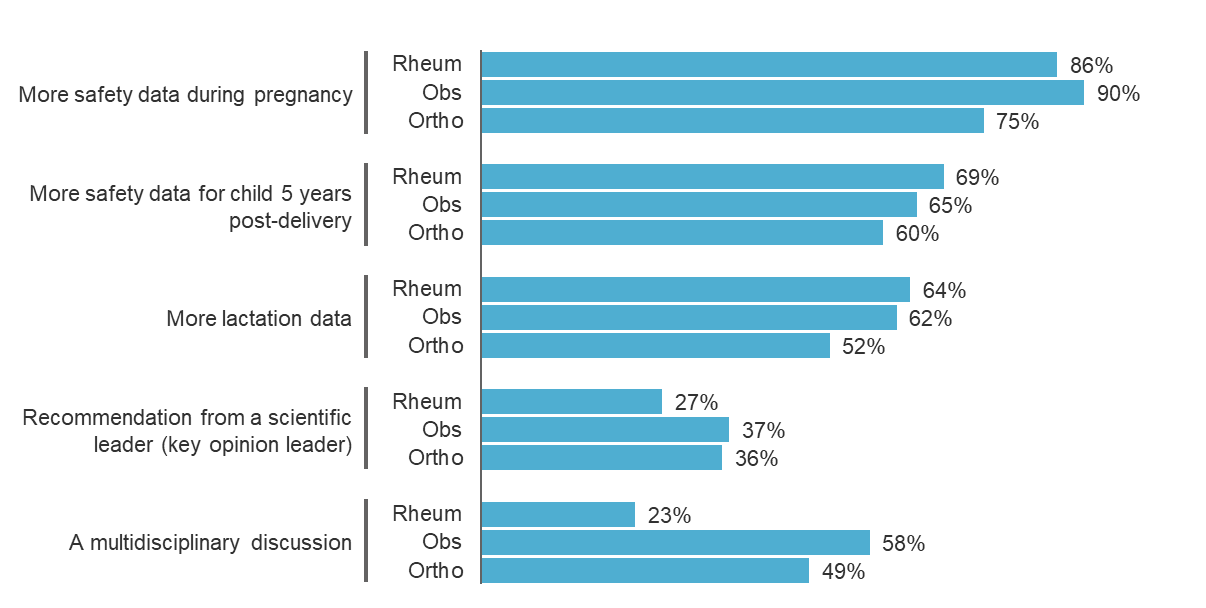


‘Hypothetically, what, if anything, would make you more comfortable with using TNFi agents among female patients between the age of 18-45 who may become pregnant in the future?’ APAC: n=335 (rheumatologists: n=174, obstetricians: n=84 and orthopaedic surgeons: n=77). Orthopaedic surgeons were included for Japan only. APAC: Asia-Pacific (Australia, Japan, Hong Kong/Taiwan); Obs: obstetricians; Ortho: orthopaedic surgeons; Rheum: rheumatologists; TNFi: tumour necrosis factor inhibitors.

Supplementary Figure 4. Plain language summary


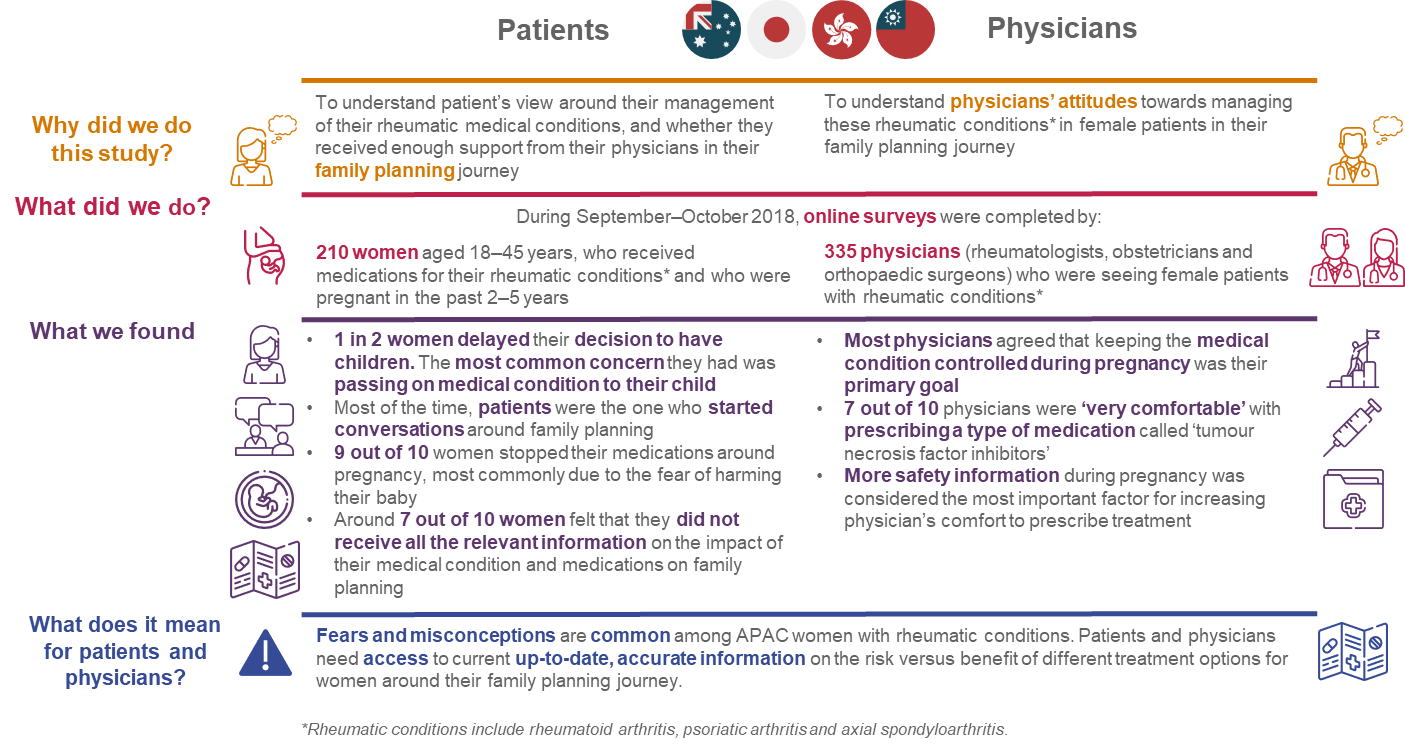

Supplement: Supplementary file 1 — Supplementary file1 (DOCX 293 KB) [file 296_2022_5206_MOESM1_ESM.docx]
